# A telephone survey of parental attitudes and behaviours regarding teenage drinking

**DOI:** 10.1186/1471-2458-10-297

**Published:** 2010-06-01

**Authors:** Bobby P Smyth, Catherine D Darker, Erica Donnelly-Swift, Joe M Barry, Shane PA Allwright

**Affiliations:** 1 Department of Public Health & Primary Care, Trinity College Dublin, Ireland

## Abstract

**Background:**

Irish teenagers demonstrate high rates of drunkenness and there has been a progressive fall in age of first drinking in recent decades. International research indicates that parents exert substantial influence over their teenager's drinking. We sought to determine the attitudes and behaviours of Irish parents towards drinking by their adolescent children.

**Methods:**

We conducted a telephone survey of a representative sample of of 234 parents who had a teenager aged between 13 and 17 years.

**Results:**

Six per cent reported that they would be unconcerned if their son or daughter was to binge drink once per month. On the issue of introducing children to alcohol in the home, 27% viewed this as a good idea while 63% disagreed with this practice. Eleven per cent of parents reported that they had given a drink to their teenager at home. Parents who drank regularly themselves, who were from higher socio-demographic groups and who lived in the east of Ireland demonstrated more permissive attitudes to teenage drinking.

**Conclusions:**

We found no evidence of widespread permissive attitudes and behaviours among Irish parents. Given that parental influences have been demonstrated to exert substantial impact on teenage drinking, it may be possible to harness the concerns of Irish parents more effectively to reverse the trends of escalating alcohol related harm in Ireland.

## Background

The third highest rate of drunkenness in Europe has been reported for Irish teenagers, with only teenagers from Britain and the Isle of Man getting drunk more frequently [1]. In the past two decades, the average age of onset of drinking has dropped dramatically in Ireland by 3.5 years to 15 years [2]. Mongan et al reported an increase in the number of children presenting to A&E for alcohol related reasons in the past decade [3].

Early onset drinking is associated with a range of negative outcomes, including increased risk of later alcohol dependence [4-7], increased risk of drug abuse [8,9], criminal behaviour [10], sexual risk behaviour [11] and suicidal behaviour [12]. Recent research has also highlighted the negative impact which regular alcohol use can have on the developing adolescent brain [13].

Although peers exert an important influence on teenage drinking [14,15], research on familial risk and protective processes provides independent support for multiple domains of parental influence on adolescent drinking [15-17]. Parental alcohol use can influence adolescents' use, with greater parental alcohol use increasing the chance of early onset of alcohol use and subsequent unhealthy drinking patterns amongst adolescents [15,17-20]. There is good evidence that the establishment of clear alcohol specific rules leads to a delay in the onset of drinking [20-22]. Some researchers have cautioned that an extremely harsh authoritarian approach to teenage alcohol use may be counterproductive [23]. In Sweden, a brief prevention program which encouraged parents to maintain strict attitudes towards teenage drinking successfully reduced drunkenness [24]. A recent meta-analysis of parent and family preventions interventions demonstrated their effectiveness in reducing adolescent alcohol problems [25].

There appears to be wide diversity in parents' beliefs about how best to encourage non-harmful drinking behaviour in their children and considerable anxiety about what is the best approach [16,26,27]. Researchers also have quite polarised views on what parents should do [18,28]. In Britain, some parents have begun to embrace what they consider to be the 'continental model' and opt to introduce children to alcohol in the home at an early age. It has been advocated by Bellis et al, based on their finding that, among teenage drinkers, those who source alcohol from parents experience less alcohol related harm than those who source their alcohol elsewhere [28]. Almost half of the 15 and 16-year-olds in Bellis' study reported sourcing alcohol from parents. On the other hand, in a British qualitative study, it emerged that children brought up in families who embrace the 'continental model' tend to drink to excess while being extremely resistant to advice about alcohol related harms [26]. Warner & White demonstrated that earlier introduction to drinking was associated with increased risk of later alcohol abuse, even if the introduction to alcohol occurred with parents [7]. In a qualitative Irish study of treatment attending teenagers with serious drug and alcohol problems, Palmer & O'Reilly found that permissive attitudes of parents towards alcohol were linked to development of later problematic substance misuse [8]. In USA, Abar et al demonstrated that university students living away from home were more likely to drink excessively and to experience alcohol related harms if they grew up in a family which permitted underage drinking [18]. Others have also found that a permissive approach to teenage drinking is associated with greater alcohol abuse [14,19]. The vast majority of research on this issue has examined parenting practices based on the report of their children. A recent comprehensive review of underage drinking concluded that there was a need to gain a better understanding of parents' actual beliefs about this issue [15].

In light of the research evidence that indicates that parental attitudes and behaviours exert very significant influence on adolescent drinking, it is unlikely that the high rates of alcohol abuse demonstrated by Irish teenagers, the marked reduction in age of alcohol initiation and self-reported ease of access to alcohol are happening without the tacit consent of Irish parents. We sought to determine from parents themselves if the provision of alcohol to children is now widely supported and practised by parents in Ireland. We also sought to examine if parental attitudes and behaviours were associated with socio-demographic characteristics or associated with the parents' own drinking behaviours.

## Methods

A telephone questionnaire survey was conducted to ascertain parental attitudes and behaviours regarding the introduction of their teenage children to alcohol in the home. The questionnaire was designed *de novo*. In addition to questions on their socio-demographic characteristics, parents were asked if they had offered their teen a drink, what type of alcohol and on what types of occasions; about their attitudes to teen drinking; and their own drinking status.

A pilot survey on a convenience sample of 17 parents of teens (aged 13 -- 17 years) was carried out by members of the research team to assess the clarity and acceptability of the questions. Only minor wording changes were required. Five (29%) of these parents said they had offered their teen a drink when they were having one. This proportion implies that a sample size of around 225 parents of teens would be required in order to estimate the proportion of Irish parents offering their teenage child an alcoholic drink within ± 6% with 95% confidence.

The survey was carried out by a commercial survey company, Ipsos MRBI. The Ipsos MRBI PhoneBus is a fortnightly telephone omnibus survey of the adult population of the Republic of Ireland aged 15 + years which aims to collect a fully representative national sample of 1,000 adults (at least 950 adults 18 + years in each wave) with census derived quota controls in terms of sex, age and region, and the Irish market research industry classification of social class (annual Joint National Readership Survey, based on census data and the quarterly national household survey estimates). Respondents were contacted for interview on both landline and mobile telephone numbers, using Random Digit Dialing. The sample includes 40% mobile phone numbers and 60% landline phone numbers. Minimum and maximum quota (+/- 10%) are set and then data are weighted to yield the exact target. The survey was conducted in accordance with stipulations laid out by the Office of the Data Protection Commissioner. It also adhered to ESOMAR, MRS and AIMRO (of which Ipsos MRBI are members) requirements for conducting market research surveys.

The questions were administered only to respondents who had children. A random sample of 632 parents was contacted by telephone between September and November 2008, yielding 234 parents of teens (13-17 years). If the respondent had more than one child who was currently aged between 13-17 years, they were asked to answer the questions with respect to the child whose birthday came next. If they did not yet have any teenagers, they responded based on what they thought they might do or think in the future.

The term 'binge drinking' refers to an episode of excessive drinking. It is generally accepted that anything in excess of six 'units' or 'standard drinks' constitutes a binge. In order to explore parents' views about their teenager engaging in binge drinking, we asked them specifically about their views on their teenager drinking four pints of beer or the equivalent, this being 8 'units' or about 80 grams of pure alcohol on a single occasion. We also sought the views of parents of how many units they thought a person had to drink for the episode to be considered a 'binge'.

The Chair of Research Ethics Committee of the Faculties of Occupational Medicine and Public Health Medicine was contacted with regard to this study. The study was granted exemption.

### Statistical analysis

Univariate analysis with the chi square test was conducted to determine predictors of Irish parents' attitudes towards teenage drinking. Odds ratios and their 95% confidence intervals were calculated.

We also determined an overall attitude score for each parent based upon their responses to the three attitude questions, namely views on provision of alcohol to their children by other adults, concern regarding binge drinking and views on the value of introducing children to alcohol in the home. For each question the response "strongly disagree" was assigned a score of zero, "slightly disagree" a score of 1, "neither agree nor disagree" a score of 2, "slightly agree" scored 3 and responses of "strongly agree" scored 4. The cumulative score ranged from zero to twelve with low scores indicating conservative attitudes and high scores indicating permissive views on teenage drinking. As the data were highly skewed, we used the Mann Whittney U test to explore the parental characteristics associated with this overview score.

## Results

### Descriptive characteristics, behaviours and attitudes

All respondents were parents (i.e. none of the respondents were guardians). Overall, 632 parents had at least one child under 18 years of age in the household (339, 54% female and 293, 46% male). There were 234 parents with a teenager aged 13-17 years in the household. Table 1 provides descriptive statistics for parents with and without teenage children.

**Table 1:** Socio-demographic characteristics of parent respondents (n = 632)

	Parents of teens (13-17 yrs) (N = 234)		Parents of younger children (<13 yrs) (N = 398)	
	Count	%	Count	%
Gender of parent				
Female (n = 339)	118	50.4	221	55.5
Male (n = 293)	116	49.6	177	44.5
				
Age of Parent				
Under 25 yrs (n = 24)	0	0.0	24	6.0
25-34 yrs (n = 172)	20	8.5	152	38.2
35-44 yrs (n = 267)	89	38.0	178	44.7
45-54 yrs (n = 141)	103	44.0	38	9.5
Over 54 yrs (n = 28)	22	9.4	6	1.5
				
Region				
Dublin (n = 131)	56	23.9	75	18.8
Rest of Leinster (n = 206)	68	29.1	138	34.7
Munster (n = 171)	62	26.5	109	27.4
Connaught/Ulster (n = 124)	48	20.5	76	19.1
				
SEGP^				
AB (n = 88)	29	12.4	59	14.8
C1 (n = 170)	54	23.1	116	29.1
C2 (n = 185)	69	29.5	116	29.1
DE (n = 135)	55	23.5	80	20.1
F (n = 54)	27	11.5	27	6.8
				
Work Status				
Not Working outside of home(n = 145)	52	22.2	93	23.4
Working (n = 487)	182	77.8	305	76.6
				
Parents' drinking status*				
Regular# (n = 123)	50	21.4	73	18.5
Infrequent (n = 401)	149	63.7	252	64.0
None (n = 104)	35	15.0	69	17.5
				
Parent offering their teenager an alcoholic drink				
No (n = 209)	209	89.3	NA	NA
Yes (n = 25)	25	10.7	NA	NA
				
Parent classification of binge drinking*				
4-5 units (n = 91)	28	12.0	63	16.0
6 units (n = 211)	77	32.9	134	34.0
7-8 units (n = 209)	86	36.8	123	31.2
8+ units (n = 62)	20	8.5	42	10.7
Don't know (n = 55)	23	9.8	32	8.1

^ SEGP classification: AB: upper & middle class; C1: lower middle class; C2: skilled working class; DE: unskilled working class; F: farmers

* There were four refusals/unknown responses.

# Regular drinkers consumed alcohol at least twice per week. Infrequent drinkers consumed alcohol once per week or less frequently.

### Provision of alcohol to children by parents

Only 25 (11%) of parents of teens said they had offered their teen an alcoholic drink. The modal age at which this happened was 16 years (n = 10); four of these 25 parents gave their child a drink prior to their 15^th ^birthday. Most (19) of these parents provided the first alcoholic drink at a special occasion. Parents who provided drink to their teenagers usually provided beer (9) or wine (7).

Table 2 shows the parents' responses to the three attitude questions. Parents of teens were more likely to disagree with these three statements than parents of younger children.

**Table 2:** Attitudes of parent respondents towards teenage alcohol consumption (n = 628*)

	*"It would be OK for another parent/guardian to provide my son/daughter with alcohol under supervision."*				*"I would not be concerned if my teenager drank four pints of beer (or equivalent) once a month."*				*"It's a good idea to introduce teenagers under 18 to alcohol in the home."*			
	**Parents of teens (13-17 yrs)**		**Parents of younger children (<13 yrs)**		**Parents of teens (13-17 yrs)**		**Parents of younger children (<13 yrs)**		**Parents of teens (13-17 yrs)**		**Parents of younger children (<13 yrs)**	
	**(N = 234 ) (%)**		(N = 394)* (%)		**(N = 234) (%)**		(N = 394)* (%)		**(N = 234) (%)**		(N = 394)* (%)	

												
**Strongly Agree**	7	(3.0)	24	(6.1)	9	(3.8)	29	(7.4)	25	(10.7)	45	(11.4)
												
**Slightly Agree**	10	(4.3)	15	(3.8)	5	(2.1)	28	(7.1)	37	(15.8)	68	(17.3)
												
**Neither**	6	(2.6)	12	(3.0)	9	(3.8)	34	(8.6)	24	(10.3)	68	(17.3)
												
**Slightly Disagree**	9	(3.8)	18	(4.6)	17	(7.3)	47	(11.9)	25	(10.7)	33	(8.4)
												
**Strongly Disagree**	202	(86.3)	325	(82.5)	194	(82.9)	253	(64.2)	123	(52.6)	180	(45.7)
												
**Don't Know**	0	(0.0)	0	(0.0)	0	(0.0)	3	(0.8)	0	(0.0)	0	(0.0)

*There were four refusals/unknown responses.

Ninety per cent (211) of the parents of teenagers disagreed with the statement that it would be OK for another parent to give their teenager a drink. Parental response to this item was not associated with socio-demographic characteristics or with parental drinking frequency. Parents who had offered their son/daughter an alcoholic drink whilst they themselves were having a drink were significantly less likely to disagree with this statement than those who had not offered their teenager a drink (odds ratio [OR] 0.13, 95%CI= 0.04-0.39) and parents of teenagers aged 17 years were significantly less likely to disagree than parents of teenagers aged 13 years (OR 0.07, 95% C.I. = 0.01, 0.62). .

### Concern about binge drinking

Two hundred and eleven parents of teens (90%) disagreed with the statement "I would not be concerned if my son or daughter drank four pints of beer (or equivalent) once a month". No parental or teenager characteristic was significantly associated with response to this item.

### Introducing children to alcohol at home

There was more divergence of opinion on the issue of introducing children to alcohol in the home with 27% of parents of teens agreeing that this was a good idea, 63% (148) disagreeing and 10% being unsure. The characteristics of parents of teenagers who disagreed with this notion relative to parents who agreed that it was a good idea are presented in table 3. Parents of teenagers who drank infrequently (OR 2.6) or not at all (OR 10), who were from the western and northern provinces of Connaught and Ulster (OR 4.0), or from lower socio-economic groups were significantly more likely to disagree with this idea. Parents who were working (OR 0.4) and those who said they had offered their teen a drink (OR 0.1) were significantly less likely to disagree (i.e. more likely to believe that it was a good idea to introduce children to alcohol in the home). There was a trend toward increased support for this idea among parents with more liberal definitions of binge drinking.

**Table 3:** Factors associated with parents of teenagers disagreeing with the statement that "It's a good idea to introduce teenagers under 18 to alcohol in the home"*

	Odds Ratio	95% C.I.	P-value
Gender of parent			
Female	1.0		
Male	0.9	(0.5, 1.6)	NS
			
Age of Parent			
25-34 yrs	0.9	(0.3,2.7)	NS
35-44 yrs	1.0		
45-54 yrs	0.7	(0.3,1.3)	NS
Over 54 yrs	0.7	(0.2,1.9)	NS
			
Region			
Dublin	1.0		
Rest of Leinster	1.7	(0.7,3.7)	NS
Munster	1.8	(0.8,4.0)	NS
Connaught/Ulster	4.0	(1.5,10.8)	<0.05
			
SEGP^			
AB	1.0		
C1	2.0	(0.7, 5.4)	NS
C2	2.7	(1.0, 7.3)	<0.05
DE	5.6	(1.8, 7.0)	<0.01
F	3.3	(0.9,11.2)	0.06
			
Work Status			
Not Working outside of home	1.0		
Working	0.4	(0.1, 0.9)	<0.05
			
Parents' drinking status#			
Regular	1.0		
Infrequent	2.6	(1.3, 5.2)	<0.01
None	10.0	(2.6, 37.9)	<0.01
			
Parent had given their teenager an alcoholic drink			
No	1.0		
Yes	0.1	(0.0,0.4)	<0.01
			
Parent classification of binge drinking			
4-5 units	0.9	(0.3, 2.4)	NS
6 units	1.0		
7-8 units	0.9	(0.4, 1.8)	NS
8 + units	0.5	(0.2, 1.4)	NS
Don't know	2.6	(0.7, 9.9)	NS
			
Age of teenager			
13 years	1.0		
14 years	0.6	(0.2, 1.5)	NS
15 years	0.4	(0.2,1.1)	NS
16 years	0.6	(0.2,1.6)	NS
17 years	0.4	(0.4, 1.2)	NS

* 148 parents who disagreed with the statement compared with 62 parents who agreed with the statement; 24 parents in the 'neither' category excluded.

^ SEGP classification: AB: upper & middle class; C1: lower middle class; C2: skilled working class; DE: unskilled working class; F: farmers

# Regular drinkers consumed alcohol at least twice per week. Infrequent drinkers consumed alcohol once per week or less frequently.

Table 4 presents a cumulative score indicating how permissive a parent was in terms of their attitudes to teenage drinking. Scores ranged from zero to 12, with a median of 1 and a mean of 1.9. Parents in the eastern province (Leinster, including Dublin) and parents from the two highest socio-economic groups (AB) were significantly more permissive (higher scores) than any of the other groups. Regular drinkers were significantly more permissive than infrequent or non-drinking parents and parents who had given alcohol to their children in the past were significantly more permissive than those who had not done so. The Cronbach alpha score for the reliability of this scale was 0.53.

**Table 4:** Cumulative score for attitudes of 234 parents towards their teenager's drinking, higher score indicative of more permissive attitudes.

	number	mean	median	Inter Quartile Range	p value*
Total group	234	1.9	1	0 - 3	
					
Gender of parent					
Female	116	1.9	1	0 - 3	
Male	118	1.9	1	0 - 3	NS
					
Age of parent					
Under 45 yrs	109	1.7	1	0 - 3	
45 yrs and over	125	2.1	1	0 - 4	NS
					
Location					
Dublin	56	2.3	2	0 - 4	No sig diff between Dub, RoL & Muns
Rest of Leinster (RoL)	68	2.3	1	0 - 3	
Munster	62	1.7	1	0 - 3	Con vs Dub, <0.01 Con vs RoL, 0.04
Connaught/Ulster	48	1.3	0	0 - 2	Con vs Munster NS
					
Socio-Economic Group					
AB	29	2.7	3	1 - 4	
C	123	1.9	1	0 - 3	AB vs C, 0.03
DE	55	1.6	1	0 - 2	AB vs DE, 0.004
F	27	1.6	0	0 - 3	AB vs F, 0.02
					
Parents drinking status#					
Regular	50	2.8	2	0 - 4	
Infrequent	149	1.7	1	0 - 3	reg vs infreq 0.02
None	35	1.4	0	0 - 2	non vs reg 0.003 infreq vs non, NS
					
Parent had given their teenager an alcoholic drink					
Yes	25	4.5	4	3 - 6	
No	209	1.6	1	0 - 3	<0.001
					

* Mann-Whitney U test

# Regular drinkers consumed alcohol at least twice per week. Infrequent drinkers consumed alcohol once per week or less frequently.

## Discussion

This survey provides the first information on how parents in Ireland treat the issue of introducing alcohol to teens in the home. Among the parents with teenagers, just 11% reported that they had given alcohol to their son or daughter in the family context. Although Bellis et al examined a narrower age range and surveyed teenagers rather than parents, it appears that this behaviour is much more common in Britain [28]. Research from Sweden and New Zealand, has found that the vast majority of parents report a restrictive attitude towards supplying their teenagers with alcohol [24,29]. Where Irish parents provided alcohol, this occurred at age 16 or 17 in the majority of cases, and less than 2% of all parents had given their teenager alcohol before the age of 15 years. A similar pattern has also been found in Australia and New Zealand [16,29]. Our findings indicate that the progressive fall in age of first drinking in Ireland cannot be explained by widespread introduction of children to alcohol by parents. We know that drinking typically commences in Ireland now at age 14 or 15 years [2]. Hence, it is likely that many of the children who were 'introduced' by their parents to alcohol at home, were in fact already drinking for one or two years outside of home.

Just over a quarter of parents thought that it was a good idea to introduce their children to alcohol. This issue divided parents, with almost half strongly opposing this practice. A study in New Jersey by Warner & White found that introduction to alcohol at a younger age, even if done at home, was predictive of later alcohol problems [7]. It is of concern that the parents who drank most regularly were more likely to embrace this idea and this mirrors findings from British research [26]. As in Britain, it appears that some Irish parents have attempted to introduce 'continental style' drinking to Irish homes in the hope that it will divert their teenage children away from 'binge' or heavy episodic drinking [26]. American research has highlighted the counterproductive impact of such approaches [18]. Where the pervasive culture is highly tolerant of heavy drinking, as it is in Britain and Ireland, early drinking is linked to later alcohol abuse [8,22].

The vast majority of Irish parents would not permit another parent to give alcohol to their child. Also, around 90% of parents indicated that they would be concerned if their teenage son or daughter was binge drinking once a month, with only 6% being unconcerned by this behaviour. It is difficult to reconcile these findings with the very high rates of drunkenness and the frequent purchasing of alcohol reported by Irish teenagers [1,8]. This suggests that many Irish parents exert little control over their teenage children's behaviour, or else they have been persuaded to accept regular drunkenness, despite their own concerns and unease about this behaviour. Research from Britain, Australia and Ireland supports this latter possibility with many parents reporting being confused about what to do and many feeling disempowered in addressing this issue [16,26,27]. International research indicates that parents consistently underestimate their children's drinking [16,29].

In terms of socio-demographic trends, there was a tendency for parents from Dublin to have more liberal views than those in more rural locations and the lower socio-economic group were the most conservative. In Britain it was also those from professional classes who were most inclined to provide alcohol to children at home [26]. In contrast, research from the Netherlands indicated that parents from higher socio-economic groups were stricter about alcohol [19]. They also found that being permissive about alcohol use was linked to greater alcohol abuse in teenagers, and that this effect was most pronounced in children from higher socio-economic groups. Our findings highlight challenges in terms of public policy formation, as those who exert most influence on policy in Ireland, including health professionals, politicians and journalists, belong to the permissive class on this issue [26].

The limitations of this study relate to the use of a non-validated questionnaire and that, although the sample is representative of the Irish population, it is modest in size. The reliability of the permissiveness score generated by combining responses to the three key issues explored was suboptimal, with a Cronbach alpha of 0.53, which is lower than the rule of thumb reliability of 0.70. This compromises its interpretation. However, it was designed to be as short as possible which necessarily compromised its reliability. The significant associations found between permissive attitudes and geographic region may possibly be explained by variations in work status and socio-economic group. Unlike most previous research on this topic, we focused on the views of parents, rather than on the teenagers' perceptions of their parents' attitudes. Conducting a matched interview with their teenage sons and daughters and exploring their actual alcohol use and associated harms, would have added to the study, but was not practicable. Interviews were conducted via telephone and responses may have been different if face to face interviews had occurred. For example, telephone interviews have been shown to elicit higher levels of personal experience of alcohol related harms [30]. Although it is not illegal for parents to give alcohol to their children, it is a contentious topic and social desirability may have caused parents to under-report this behaviour [31].

## Conclusions

Our findings do not lend support to a view that there is widespread complacency about teenage drinking among Irish parents. They are concerned and generally quite conservative about this issue. Hence, rather than being viewed as part of the problem, our findings suggest that parents represent a largely untapped resource and can be part of the solution in reversing the worrying trends of the past couple of decades.

## Competing interests

The authors declare that they have no competing interests.

## Authors' contributions

BS, SA, CD & JB conceived the study and participated in its design. SA, CD, JB & BS designed the questionnaire and conducted the pilot study. SA coordinated the study. ED-S conducted the statistical analysis. BS took the lead on drafting the manuscript. All authors contributed to drafting of the manuscript and all have read and approved the final manuscript.

## Pre-publication history

The pre-publication history for this paper can be accessed here:

http://www.biomedcentral.com/1471-2458/10/297/prepub
